# Clinical analysis of secondary glaucoma in Central China

**DOI:** 10.1038/s41598-023-34872-8

**Published:** 2023-05-25

**Authors:** Qian Liu, Changgeng Liu, Wenjun Cheng, Xiaomei Feng, Haijun Li, Xiaoyuan Yang, Yangzeng Dong

**Affiliations:** Henan Provincial People’s Hospital, Henan Eye Hospital, Henan Eye Institute, Zhengzhou University People’s Hospital, No. 7 Weiwu Road, Zhengzhou, 450003 Henan China

**Keywords:** Eye diseases, Medical research

## Abstract

To describe the epidemiology, clinical and social characteristics, management, and outcomes of patients with secondary glaucoma in Central China, a total of 1,129 cases (1,158 eyes) among 710 males (62.89%) and 419 females (37.11%) were analyzed. The mean age was 53.75 ± 17.11 years. The New Rural Cooperative Medical System (NCMS) played the most important role in reimbursement (60.32%) for secondary glaucoma-related medical expenses. The predominant occupation was “farmer” (53.41%). Neovascularization and trauma were the leading causes of secondary glaucoma. Cases of trauma-induced glaucoma decreased substantially during the coronavirus disease 2019 (COVID-19) pandemic. An education level of senior high school or above was uncommon. Ahmed glaucoma valve implantation was the most commonly performed surgery. At the final follow-up, the overall intraocular pressure (IOP) in patients with vascular disease- and trauma-related secondary glaucoma was 19.53 ± 10.20 mmHg, 20.26 ± 11.75 mmHg, and 16.90 ± 6.72 mmHg, while the mean visual acuity (VA) was 0.33 ± 0.32, 0.34 ± 0.36, and 0.43 ± 0.36. In 814 (70.29%) eyes, the VA was < 0.01. Effective preventive measures for at-risk populations, increased NCMS coverage and the promotion of higher education are necessary. These findings will help ophthalmologists detect secondary glaucoma early and manage it in a timely manner.

## Introduction

Secondary glaucoma is a group of eye diseases caused by ocular disorders or systemic factors that affect aqueous humor drainage, further resulting in elevated intraocular pressure (IOP) and visual function impairment^1–3^. Secondary glaucoma occurs when a second form of ocular pathology causes IOP elevation, leading to optic nerve and visual field damage^4^. Common secondary ocular pathological processes include trauma, neovascularization uveitis, surgery- and lens-induced issues, drug-related issues, and tumors^4, 5^. Secondary glaucoma is an important risk factor for acquired irreversible blindness^2^.

Surgery is the mainstay for treating secondary glaucoma due to the complications associated with secondary factors^5^. The prevalence and recovery rate of secondary glaucoma largely depend on education level, the local economy, and the level of medical services available^3^. In China, Henan Province has the third largest population. This area is a major agricultural region that is home to numerous farm workers from Central China. Many people who lack education regarding occupational protection are vocationally engaged in the agriculture industry, which increases their risk of secondary glaucoma. The available medical services in Henan Province are unevenly distributed, which increases the risk of blindness due to secondary glaucoma. Henan Eye Hospital is one of the leading domestic eye hospitals in Central China that annually services a large number of secondary glaucoma patients from remote areas or areas with low levels of medical care. These circumstances may indicate that the likelihood of blindness due to secondary glaucoma is higher in this province than in more economically developed coastal areas of China. The coronavirus disease 2019 (COVID-19) pandemic has exacerbated this situation. Against this background, the promotion and popularization of glaucoma prevention is particularly necessary and is a low-cost, effective method.

Secondary glaucoma is usually a complicated condition that can easily result in blindness; thus, it is a serious public health concern in Henan. Low economic levels, unevenly distributed medical services and a lack of prevention communication may increase the risk of blindness due to secondary glaucoma. Summarizing and exploring the characteristics, epidemiology, management and outcomes of secondary glaucoma may be potentially important for the prevention of blindness due to secondary glaucoma in developing countries and regions. To date, no large studies have examined the characteristics or outcomes of secondary glaucoma in Central China. Therefore, we analyzed the characteristics of secondary glaucoma in patients who visited the ophthalmology departments at Henan Eye Hospital, Henan Eye Institute, and Henan Provincial People’s Hospital.

## Methods

This retrospective study was approved by the Ethics Committees of Henan Eye Hospital and Henan Eye Institute and the Henan Provincial People’s Hospital Human Research Ethics Committee (approval number HNEECKY-2022(08)). This study adhered to the tenets of the Declaration of Helsinki. Informed consent was obtained from all patients and/or their guardians.

We retrospectively reviewed the data of all inpatients (n = 7,668) who were admitted and treated at Henan Provincial People’s Hospital, Henan Eye Hospital and Henan Eye Institute between 2017 and 2021. These centers have good reputations in Henan and Central China and receive patient referrals for complicated cases of glaucoma from hospitals in remote regions and county-level and city-level tertiary hospitals for diagnoses. The composition of patients who visit these centers is representative of patients from across Central China. The clinical and social characteristics of patients, including age, sex, medical insurance, occupation, educational background, IOP, visual acuity (VA), medications, surgical options, and outcomes, were analyzed.

Patients with secondary glaucoma in this study were identified by a qualified glaucoma specialist. Secondary glaucoma was properly considered to represent eyes in which a second form of ocular pathology caused IOP elevation, leading to optic nerve damage^4^. The secondary ocular pathological process included one of the following: trauma, lens-related issues, uveitis, neovascularization, intraocular surgery and others^5^. Surgery was performed in cases of primary disease requiring treatment, if progression of glaucomatous damage was demonstrated, or if, in the surgeon’s opinion, the IOP was at a level that would cause additional damage. VA was documented according to the standard decimal VA chart. IOP was measured using an iCare tonometer (iCare, Vantaa, Finland) and noncontact tonometer (Canon, Ota, Japan). Patients who were admitted for reasons not primarily related to glaucoma treatment were excluded. Patients with records that documented a history of primary glaucoma were excluded from the analysis.

### Statistical analysis

Data were analyzed using SPSS software (version 19.0.0; IBM Corp., Armonk, NY, USA). Clinical and social characteristics were analyzed before and after the COVID-19 pandemic. Continuous and categorical variables are presented as the means ± standard deviations and percentages, respectively. Ages were compared by *t* tests. Pearson’s χ^2^ test was used to compare patients with different etiologies, social characteristics, and treatment composition ratios before and after the COVID-19 pandemic. *P* < 0.05 was considered statistically significant.

## Results

### Secondary glaucoma characteristics and epidemiological findings

During the 5-year study period, 7,668 glaucoma charts were reviewed, and the data of 1,129 patients with secondary glaucoma (1158 eyes), comprising 710 males (62.89%) and 419 females (37.11%), were analyzed (Table 1). Twenty-nine patients had bilateral secondary glaucoma. The male–female ratio was 1.69. The mean age was 53.75 ± 17.11 years. Among male patients, the highest incidence of secondary glaucoma was found in the middle-aged and older age groups (40–49, 50–59, 60–69 years). Among female patients, the highest incidence was found in older age groups (50–59, 60–69, 70–79 years) (Supplementary S1).

**Table 1:** Secondary Glaucoma Patient and Ocular Characteristics.

Characteristics	Total* (N = 1129 patients and 1158 eyes)
Age (at time of surgery)*	
Mean (standard deviation)	53.75 (17.11)
Gender	
Male	62.89% (710)
Female	37.11% (419)
Number of eyes in study	
1	1129
2	29
Prior surgery	
Glaucoma surgery	44
Trabeculectomy (Trab)	21
Ahmed glaucoma valve implantation	15
Endoscopic cyclophotocoagulation (ECP)	5
Non-penetrating trabeculectomy	3
Non-glaucoma surgery	674
Vitrectomy	311
Phacoemulsification	126
Intravitreous anti-VEGF injection	117
Silicone oil extraction	66
Suture of ocular trauma	30
Penetrating Keratoplasty	16
Intraocular lens extraction	1
Others	7
Prior numbers of glaucoma medications	
1	283
2	121
3	85
4	46
Uncertain	59

*For the 29 patients with both eyes included in the study.

Overall, glaucoma surgery was performed for 44 eyes, and nonglaucoma surgery was performed for 674 eyes before admission to Henan Eye Hospital. Vitrectomy was the most common form of surgery. Overall, 594 eyes (51.30%) were treated with glaucoma medications before the patients visited Henan Eye Hospital. Of the 594 eyes, 252 (42.42%) were treated with more than one medication for lowering IOP (Table 1 and Supplementary S1).

### Social characteristics of secondary glaucoma patients

There was no significant difference in the mean age of the patients with secondary glaucoma between the period before and after the COVID-19 pandemic (2017–2019 vs. 2020–2021). Overall, 459 (62.70%) males and 273 (37.3%) females were included during 2017–2019. There was no significant change in the male–female ratio between the period before and after the COVID-19 pandemic.

The medical insurance, occupation, and educational background characteristics are listed in Table 2. The New Rural Cooperative Medical System (NCMS) played the most important role in reimbursement (60.32%) for medical expenses related to secondary glaucoma in Henan, Central China. More than half of the patients (58.88% before and 62.97% after the COVID-19 pandemic) benefited from reimbursements from the NCMS. Employee-based basic medical insurance (UE-BMI) (19.22%) and urban resident basic medical insurance (UR-BMI) (11.07%) were other important components of basic medical insurance coverage. The ratio of self-paying patients significantly decreased during 2020–2021 (*p* = 0.02).

**Table 2:** Medical insurance, education and occupations findings in patients with surgical secondary glaucoma*.

Characteristic	2017–2021 (N,%) *	2017–2019 (N,%) *	2020–2021 (N,%)*#	*P* value	
Age					
(Mean, standard deviation)	53.75 (17.11)	52.43 (16.00)	54.49 (17.66)	0.04^§^	
Gender					
Male	710 (62.89%)	459 (62.70%)	251 (63.22%)	0.86	
Female	419 (37.11%)	273 (37.30%)	146 (36.78%)	0.94	
Medical insurance					
NCMS	681 (60.32%)	431 (58.88%)	250 (62.97%)	0.18	
UE-BMI	217 (19.22%)	134 (18.31%)	83 (20.91%)	0.29	
UR-BMI	125 (11.07%)	87 (11.89%)	38 (9.57%)	0.24	
Self-paying	67 (5.93%)	52 (7.10%)	15 (3.78%)	0.02^§^	
Municipal Medical Insurance	17 (1.51%)	11 (1.50%)	6 (1.51%)	0.99	
Provincial Medical Insurance	15 (1.33%)	11 (1.50%)	4 (1.01%)	0.49	
Commercial insurance	2 (0.18%)	2 (0.27%)	0 (0.00%)	0.30	
Others or undefined	5 (0.44%)	4 (0.55%)	1 (0.25%)	0.47	
Occupations					
Farmer	603 (53.41%)	353 (48.22%)	250 (62.97%)	< 0.0001^§^	
Retirees	128 (11.34%)	84 (11.47%)	44 (11.08%)	0.84	
Staff	43 (3.80%)	25 (3.42%)	18 (4.53%)	0.36	
Student	39 (3.45%)	24 (3.28%)	15 (3.78%)	0.66	
Worker	37 (3.28%)	26 (3.55%)	11 (2.77%)	0.48	
Freelancer	35 (3.10%)	22 (3.01%)	13 (3.28%)	0.80	
Technicist	28 (2.48%)	15 (2.05%)	13 (3.28%)	0.20	
Civil Servant	22 (1.95%)	13 (1.78%)	9 (2.27%)	0.57	
Self-employed	16 (1.42%)	10 (1.37%)	6 (1.51%)	0.85	
Managers	2 (0.18%)	2 (0.27%)	0 (0.0%)	0.30	
None	40 (3.54%)	24 (3.28%)	16 (4.03%)	0.52	
Others or undefined	136 (12.05)	134 (18.30%)	2 (0.50%)	< 0.0001^§^	
Education background					
Illiteracy	89 (7.88%)	63 (8.61%)	26 (6.55%)	0.22	
Preschool	11 (0.97%)	6 (0.82%)	5 (1.26%%)	0.47	
Primary school	323 (28.61%)	209 (28.55%)	114 (28.71%)	0.95	
Junior high school	327 (28.96%)	218 (29.78%)	109 (27.46%)	0.41	
Technical secondary school	33 (2.92%)	23 (3.14%)	10 (2.52%)	0.56	
Senior high school	127 (11.25%)	82 (11.20%)	45 (11.34%)	0.94	
Junior college	88 (7.80%)	57 (7.79%)	31 (7.81%)	0.99	
Bachelor	57 (5.05%)	34 (4.64%)	23 (5.79%)	0.39	
Master	2 (0.18%)	1 (0.14%)	1 (0.25%)	0.68	
Others or undefined	72 (6.38%)	39 (5.33%)	33 (8.31%)	0.05	

*Data are shown as NO. (%).

^#^ Epidemiological analysis during Covid-19 pandemic period in China.

NCMS: New rural cooperative medical system; UE-BMI: urban employee-based basic medical insurance; UR-BMI: urban resident basic medical insurance.

^§^*p* < 0.05considered to be statistically significant.

Henan is one of the most populous provinces in China and is also a major agricultural province. “Farmer” was the predominant occupation of the patients and accounted for 53.41% of the secondary glaucoma cases in this study. The proportion of secondary glaucoma cases among farmers increased from 48.22% to 62.97% after the pandemic. The proportion of retirees (11.34%) did not change substantially between the period before (11.47%) and the period after (11.08%) the pandemic. The highest educational levels among the patients with secondary glaucoma were primary school (28.61%) and junior high school (28.96%). Secondary glaucoma patients with an educational level of senior high school or above were very rare (Table 2).

### Causes of secondary glaucoma in Central China

In the past 5 years, the most common cause of secondary glaucoma was neovascularization (43.26%), both before (42.95%) and after (43.81%) the COVID-19 pandemic (Table 3). Trauma (17.27%), posterior segment surgery (16.75%), lens-related issues (7.60%), and inflammation (4.84%) were other common causes of secondary glaucoma (Table 3). The incidence of trauma-induced glaucoma significantly decreased during the COVID-19 pandemic (2020–2021) (*p* = 0.03). The incidence of glaucoma related to posterior segment surgery with or without silicone oil tamponade increased after the COVID-19 pandemic (*p* = 0.02).

**Table 3:** Different main causes of the secondary glaucoma.

Characteristic	2017–2021 (N,%) *	2017–2019 (N,%) *	2020–2021 (N,%)*#	*P* value
Neovascularization	501 (43.26%)	317 (42.95%)	184 (43.81%)	0.78
Trauma	200 (17.27%)	141 (19.10%)	59 (14.05%)	0.03^§^
Posterior segment surgery	194 (16.75%)	110 (14.90%)	84 (20.0%)	0.02^§^
Lens-related	88 (7.60%)	62 (8.40%)	26 (6.19%)	0.18
Inflammation (uveitic)	56 (4.84%)	29 (3.93%)	27 (6.43%)	0.06
Anterior segment surgery	21 (1.81%)	17 (2.30%)	4 (0.95%)	0.09
Syndrome	8 (0.69%)	5 (0.68%)	3 (0.71%)	0.95
Intraocular tumor	7 (0.61%)	5 (0.68%)	2 (0.48%)	0.68
Drug-induced	4 (0.35%)	3 (0.41%)	1 (0.24%)	0.64
Infection	3 (0.26%)	3 (0.41%)	0 (0.00%)	0.20
Others	76 (6.56%)	46 (6.24%)	30 (7.14%)	0.55

*Data are shown as NO. (%).

^#^ Epidemiological analysis during Covid-19 pandemic period in China.

^§^*p* < 0.05considered to be statistically significant.

In the present study, neovascularization and trauma were the two leading causes of secondary glaucoma in Central China. To analyze the changes in social characteristics according to these main causes of secondary glaucoma before and after the COVID-19 pandemic, we compared neovascularization- and trauma-related cases during 2017–2019 and 2020–2021. Regarding neovascularization-related glaucoma, NCMS reimbursement increased significantly, while UR-BMI reimbursement decreased after the COVID-19 pandemic (Supplementary S2a). However, there were no significant differences in medical insurance for trauma-related glaucoma between the period before and the period after the pandemic (Supplementary S3a). “Farmer” was the most common occupation among both neovascularization- and trauma-related secondary glaucoma patients (Supplementary S2B and S3B). Unemployment significantly increased among neovascularization-related glaucoma patients (*p* = 0.04) but decreased among trauma-related glaucoma patients (*p* < 0.001) after the COVID-19 pandemic. There were also reduced proportions of workers (*p* = 0.01) and technicians (*p* < 0.01) among the patients with trauma-related secondary glaucoma (Supplementary S3b). Lower education levels (bachelor’s degree or below) were common among both neovascularization- and trauma-related secondary glaucoma patients (Supplementary S1c and S3c). Compared to the prepandemic period, in the postpandemic period, the proportion of trauma-related glaucoma patients with a junior high school education level decreased (*p* < 0.001), whereas the proportion of these patients with a senior high school education level increased (*p* < 0.001).

### Signs and symptoms of secondary glaucoma

The main signs and symptoms observed in the patients in this study are summarized in Table 4. Parental concerns were included in the presentation signs. Common clinical signs observed by ophthalmologists and symptoms included impaired vision (58.37%), eye pain (25.13%), and elevated IOP (7.25%). Buphthalmos (0.35%), red eye (0.26%), hyphema (0.17%), and foreign body sensation (0.09%) were relatively infrequent. In 6.74% of the patients with secondary glaucoma, there was no obvious discomfort during follow-up after surgical treatment.

**Table 4:** Different main primary symptoms and signs of the secondary glaucoma patients*.

Characteristic	N(number of eyes)	%
Signs and symptoms		
Impaired vision	676	58.37
Eye pain	291	25.13
Elevated IOP	84	7.25
Buphthalmos	4	0.35
Red eye	3	0.26
Hyphema	2	0.17
Foreign body sensation	1	0.09
Others		
Uncertain	19	1.64
^#^Follow-up	78	6.74

Intraocular pressure: IOP; C/D: cup/disc raito.

*Data are show as NO. (%).

^#^ Diagnosis was made at follow-ups.

### Interventions for secondary glaucoma

Surgical treatment for secondary glaucoma varied depending on the different causes. After evaluation by a glaucoma specialist in our center, the patients underwent glaucoma surgery as follows: 40.85% underwent Ahmed glaucoma valve implantation, 9.84% underwent trabeculectomy, 3.80% underwent cyclocryotherapy, 3.02% underwent endoscopic cyclophotocoagulation, 0.86% underwent trabeculotomy, 0.86% underwent nonpenetrating trabeculectomy, and 0.09% underwent EX-PRESS® Glaucoma Filtration Device implantation and ultrasound cycloplasty (Table 5). In eyes with lens-related glaucoma, 8.12% were treated with phacoemulsification, and 0.17% were treated with intraocular lens (IOL) repositioning (Table 5). Vitrectomy (11.83%) and silicone oil removal (12.44%) were performed for some patients with posterior segment diseases or trauma-related glaucoma (Table 5). Paracentesis and irrigation (0.43%) were performed in patients with anterior chamber hyphema. Sixty-two eyes were treated with ophthalmectomy, including 10 with trauma-related glaucoma, 42 with posterior segment disease-related glaucoma, three with keratitis-related glaucoma, four with retinoblastoma-related glaucoma, two with staphyloma-related glaucoma, and one with endophthalmitis-induced glaucoma (Table 5). Cataract surgery was increasingly used as secondary glaucoma treatment (10.24% vs. 6.91%) after the COVID-19 pandemic (Table 5).

**Table 5:** Management of secondary glaucoma patients*.

Characteristic	2017–2021	2017–2019	2020–2021	*P* value
	N (%)	N (%)	N (%)	
Ahmed glaucoma valve implantation	473 (40.85)	313 (42.41)	160 (38.10)	0.16
Removal of silicone	144 (12.44)	88 (11.92)	56 (13.33)	0.49
Vitrectomy	137 (11.83)	90 (12.19)	47 (11.19)	0.62
Trabeculectomy (Trab)	114 (9.84)	81 (10.98)	33 (7.86)	0.09
Phacoemulsification	94 (8.12)	51 (6.91)	43 (10.24)	0.05^§^
Evisceration of the eye	62 (5.35)	34 (4.61)	28 (6.67)	0.16
Cyclocryotherapy	44 (3.80)	24 (3.25)	20 (4.76)	0.20
Endoscopic cyclophotocoagulation (ECP)	35 (3.02)	29 (3.93)	6 (1.43)	0.02^§^
Trabeculotomy	10 (0.86)	2 (0.27)	8 (1.90)	0.71
Non-penetrating trabeculectomy	10 (0.86)	8 (1.08)	2 (0.48)	0.29
Paracentesis and irrigation for hyphema	5 (0.43)	2 (0.27)	3 (0.71)	0.28
Keratoplasty	3 (0.26)	0 (0.00)	3 (0.71)	0.02^§^
Intraocular lens reposition	2 (0.17)	2 (0.27)	0 (0.00)	0.30
ExPress glaucoma filtration device	1 (0.09)	1 (0.14)	0 (0.00)	0.46
Ultrasound Cycloplasty (UCP)	1 (0.09)	1 (0.14)	0 (0.00)	0.46
Others	23 (1.99)	12 (1.63)	11 (2.62)	0.25

*Data are shown as NO. (%)

N: Numbers of eyes

§*p* < 0.05considered to be statistically significant.

### Follow-up findings among patients with secondary glaucoma

The clinical parameters of the different types of glaucoma are described in Table 6. In all patients, the IOP decreased to 19.53 ± 10.20 mmHg at the final follow-up (Table 6). Trauma-related secondary glaucoma manifested at an earlier age than neovascularization-related secondary glaucoma (Table 6). Among 501 eyes with neovascularization-related glaucoma, the IOP decreased from 39.74 ± 12.56 to 20.26 ± 11.75 mmHg at the final follow-up. The IOP of patients with trauma-related glaucoma decreased from 36.60 ± 12.33 to 16.90 ± 6.72 mmHg at the final follow-up. The respective VA scores of all patients and those with neovascularization-related and trauma-related secondary glaucoma were as follows: 231 (19.95%), 148 (29.54%), and 10 (5.00%) eyes had no light perception at the final follow-up; 178 (15.37%), 25 (12.50%), and 88 (17.56%) eyes had light perception; 286 (24.70%), 124 (24.75%), and 51 (25.50%) eyes could perceive hand movements; and 119 (10.28%), 44 (8.78%), and 13 (6.50%) eyes could perceive finger counting, respectively. The mean VA scores of the eyes in all patients and in those with neovascularization-related and trauma-related secondary glaucoma were 0.33 ± 0.32, 0.34 ± 0.36, and 0.43 ± 0.36, respectively. From 2017 to 2021, the rate of reoperation among patients with trauma-related secondary glaucoma was 4.50% (9 eyes), which was higher than that among patients with neovascularization-related glaucoma and other cases of secondary glaucoma. Among patients with posterior segment surgery-related glaucoma, 1 patient underwent 3 eye surgeries.

**Table 6:** Final clinical outcomes among different types of secondary glaucoma*.

	2017–2021	Vascular disease	Trauma related
Presentation			
Age	53.75 ± 17.11	58.26 ± 14.61	49.36 ± 18.38
IOP (mmHg)	36.37 ± 12.99	39.74 ± 12.56	36.60 ± 12.33
LogMAR visual acuity	NLP ~ 1.2, (0.24 ± 0.19)^#^	NLP ~ 1.0, (0.13 ± 0.19)^#^	NLP ~ 0.8, (0.20 ± 0.22)^#^
Final follow-ups			
IOP (mmHg)	19.53 ± 10.20	20.26 ± 11.75	16.90 ± 6.72
LogMAR visual acuity	NLP ~ 1.5, (0.33 ± 0.32) ^#^	NLP ~ 1.2 (0.34 ± 0.36) ^#^	NLP ~ 1.0, (0.43 ± 0.36) ^#^
Re-operation interventions/eye	35 (3.02%)	14 (2.79%)	9 (4.50%)

*Data are shown as mean ± standard deviation or range.

^#^ mean ± standard is from visual acuity more than 0.01 in secondary glaucoma.

## Discussion

As there is a paucity of data on the characteristics and outcomes of secondary glaucoma in Central China, we analyzed the characteristics of secondary glaucoma among patients who visited a referral hospital in Henan Province. Neovascularization and trauma were the most common causes of secondary glaucoma in Central China. The prevalence of trauma-related glaucoma significantly decreased during the lockdown period of the COVID-19 pandemic. The NCMS was the predominant source of reimbursement for medical expenses. A high incidence of secondary glaucoma was prominent among patients with lower education levels. Ahmed glaucoma valve implantation was the most common intervention used.

Glaucoma is a leading cause of irreversible blindness worldwide^6^, imposing economic, social, and family burdens. Secondary glaucoma is caused by ocular disorders or systemic factors that induce aqueous outflow impairment, resulting in elevated IOP, which then results in glaucomatous optic neuropathy and eventual blindness^5^. Irreversible vision loss is more common in underdeveloped and developing countries. In contrast to primary glaucoma, secondary glaucoma can typically be prevented, which is important for the prevention and treatment of blindness, particularly in developing countries and regions.

There are inequalities in the level of medical care and surgical proficiency between underdeveloped and developing countries and regions. Henan is an agricultural province with an imbalance in regional medical service development and limited economic development compared to other relatively developed areas in China. Patients with secondary glaucoma usually do not receive effective initial treatment; their condition can become aggravated and irreversibly impair visual function. The COVID-19 pandemic has exacerbated this situation. Against this background, the promotion and popularization of glaucoma prevention are particularly necessary and are low-cost, effective methods. To date, epidemiological investigations on secondary glaucoma in Henan Province and its surrounding regions have been inefficient. In this study, we included patients with secondary glaucoma who underwent surgery in the ophthalmology department of Henan Eye Hospital.

The average age of the patients in this study was similar to that of the patients in a study conducted by Komaratih et al. in South Asia. Haijun et al. reported an earlier mean age at presentation in their study in southern China^5^. This could be related to different etiological compositions in different regions. Trauma-related secondary glaucoma is more likely to occur in youth. The number of male patients was higher than that of female patients (62.89% vs. 37.11%; 1.69:1) in our study, which was similar to previously published results^3, 5^. The incidence of diabetes was higher among adults aged 50 years and older and among men^7^. Moreover, high-risk professions, manual labor, violent behavior, and dangerous sports might contribute to the higher rate of global injuries among males^8–14^. Here, 62.00% of eyes had previously been treated with at least one surgery, and medical treatment had been provided for 51.30% of eyes. The overall higher rate of previous surgical failures was observed among eyes with secondary glaucoma, which might be due to the limited diagnoses, imaging technologies, and surgical equipment and techniques in undeveloped rural areas, which lead to increased surgical difficulty.

With the increasing aging population in China, the medical expenses of Chinese residents have become a widespread concern. Medical insurance is a major source of Chinese medical financing and payment^15^ and is an important factor in medical cost reimbursement for older adults^16^. Basic medical insurance and commercial insurance comprise the medical insurance system in China. The basic medical insurance system covers three different types of populations in urban and rural areas and is directly managed by central and local governments^15^. Commercial medical insurance as supplementary medical insurance has developed slowly in China. In the present study, we found that the NCMS, UE-BMI, and UR-BMI were the main payment methods and were obviously effective in relieving the medical cost burden for patients with secondary glaucoma. More than half of the patients benefited from the extensive coverage of the NCMS. The principal reason for this is probably the predominance of the agricultural population in Henan. The outbreak of COVID-19 was initially reported to the World Health Organization (WHO) on December 31, 2019. By October 31, 2020, more than 45 million people were infected with the disease, and 1.2 million deaths have been reported worldwide^17^. This disease has had a prodigious influence on life and productivity. In China, we found that basic medical insurance, such as the NCMS, UE-BMI, and UR-BMI, was clearly effective in relieving the burden of medical expenses before and after the COVID-19 pandemic. The decrease in self-payment might have been due to the decline in personal income during the COVID-19 pandemic. Our results suggested the importance of improving the coverage rate and reimbursement ratio of the NCMS, which might be helpful in easing medical costs for adults with secondary glaucoma living in undeveloped or rural regions. The predominant occupations in the present study were “farmer” and “retiree”. Somewhat similar results were observed in a report from Guangdong in southern China^5^. Unlike in Guangdong, retirees were the second most common occupational description of the population in Central China^5^. The reason may be related to the location of Guangdong, which is a developed coastal area in the southeast and a major province for labor imports. Secondary glaucoma is most pronounced among individuals with lower education levels. A total of 74.60% of patients with secondary glaucoma in Henan, Central China, had an education level of senior high school or below. Education, particularly health education, might be a factor that affects the prevalence of secondary glaucoma^3, 5^.

Neovascularization was the most prevalent cause of secondary glaucoma in this study population, accounting for 43.26% of cases. Previous studies have reported various causes of secondary glaucoma^3, 5, 18^. Similar results were observed in Chinese populations by the Zhongshan Ophthalmic Center (ZOC) in southern China^5^. In the ZOC data, trauma and vascular disease were the first and second most common causes of secondary glaucoma, respectively. The ZOC also showed that the proportion of trauma-associated secondary glaucoma had significantly decreased and that the rate of vascular disease-related secondary glaucoma had increased from 2015 to 2019. Previously, we found that trauma-related glaucoma was the most common type of secondary pediatric glaucoma^19^. Different causes of secondary glaucoma have been observed in different countries and regions. Population aging and a high incidence of diabetes among adults are associated with neovascular glaucoma in China^20^. Similar results were found in North India, where the leading cause of secondary glaucoma was neovascular glaucoma^3^. Vitrectomy-related glaucoma also comprises the majority of secondary glaucoma cases in New Delhi, India^21^. Similarly, with the increased use of vitrectomy and scleral ring ligation surgeries, the proportion of posterior segment surgery-related glaucoma has increased substantially in the last 2 years in Henan, Central China. Moreover, the quarantine established to separate and restrict the movement of people during the last 2 years of the COVID-19 pandemic contributed to a decrease in the prevalence of trauma-related glaucoma in Henan, Central China. Conversely, many studies have reported a higher prevalence of syndrome-related secondary glaucoma, which might be due to the higher prevalence of pseudoexfoliation in these countries. In Japan, exfoliating glaucoma caused most cases of secondary glaucoma^22^. Lens-induced issues were the leading cause of secondary glaucoma in neighboring South Asian countries^23^. The varied results among different countries and districts suggest that secondary glaucoma causes are diverse and may be related to ethnicity, geographic factors, educational and economic levels, and medical service availability.

Glaucoma valve implantation is an effective approach for decreasing and controlling IOP over time^24, 25^. The American Glaucoma Society group showed that glaucoma valve implantation is being selected as an alternative to trabeculectomy with increasing frequency^26–28^. Glaucoma valve implantation has been widely used in refractory glaucoma cases, including neovascular-, trauma-, and vitrectomy-related secondary glaucoma cases, in many different countries and regions^26, 27, 29–31^. Here, the most common initial glaucoma intervention for each eye was trabeculectomy. The most commonly performed surgery in our center was Ahmed glaucoma valve implantation. Cataract surgery is increasingly being used at our center as an effective treatment for glaucoma. Thus, trabeculectomy remains the preferred surgical treatment for secondary glaucoma in rural or undeveloped regions in Henan, Central China. Medical resources and ophthalmological specialists are dispersed unevenly across mainland China. The uptake of glaucoma valve implantation in most district or county hospitals is low and could be due to economic and medical service level limitations. The lack of coverage for the Ahmed valve by medical insurance in some regions could be another reason. The most common surgical procedures for managing secondary glaucoma in the past 5 years in southern China have been drainage device implantation, cataract surgery, and cyclophotocoagulation^5^. Trabeculectomy remains the classic surgical method for treating secondary glaucoma in developing or undeveloped countries or districts^5, 18^. In northern India, transscleral cyclophotocoagulation has been extensively adopted as a secondary glaucoma treatment^3^.

Impaired vision was the most common symptom among secondary glaucoma patients at our center. Previously, we found that an elevated IOP was the most common reason for glaucoma in patients^19^. Overall, for 6.74% of the patients, the diagnosis was established during follow-up. The results suggested that regular follow-up is crucial to prevent visual function loss in patients with secondary glaucoma, particularly that related to trauma or neovascularization. In the present study, stable IOP reduction was observed in most analyzed eyes with secondary glaucoma. Average VA had improved in all groups of patients by the final observation. Patients with trauma were younger than those with neovascularization. Moreover, the predominant average VA score among eyes with secondary glaucoma observed at our center was < 0.01. This may be because most patients with secondary glaucoma presented to our center with an advanced disease stage and impaired vision, given that our center is a comprehensive ophthalmic center that receives referrals of complicated cases from tertiary hospitals and rural areas. Stable follow-up, early diagnosis, and effective treatment are crucial to prevent vision loss in secondary glaucoma patients.

This study had several limitations. First, the retrospective nature of this study might have led to bias. Second, no standardized management or widely established guidelines exist for secondary glaucoma. In addition, a Goldman applanation tonometer is needed to measure IOP. Patients’ comorbidities (ocular and systemic) may have affected the results. The present study was a hospital-based analysis of patients who underwent surgical treatment for secondary glaucoma. Thus, population-based epidemiological studies are needed in the future.

## Conclusion

The present study summarized the characteristics, epidemiology, social characteristics, management, and outcomes of patients with secondary glaucoma. Neovascularization and trauma were the most common causes of secondary glaucoma in Central China. Among male patients, the highest incidence of secondary glaucoma was found in the middle-aged and older age groups. The NCMS played the most important role in medical expense reimbursement for secondary glaucoma patients in Central China. The predominant occupations were “farmer” and “retiree”. A high incidence of secondary glaucoma was found among those with lower education levels. The use of traditional antiglaucoma surgery decreased, and different advanced treatments emerged. Providing effective preventive measures for at-risk populations, increasing NCMS coverage and improving the education level of the population are necessary. Follow-up should be started early and continued for an extended duration for patients with one or several factors that increase the risk of secondary glaucoma. These findings will help ophthalmologists detect secondary glaucoma early and manage it in a timely manner.

## Supplementary Information

Supplementary Figures.

## Data Availability

The original contributions presented in this study are included in this published article, and further inquiries can be directed to the corresponding author.
