# Supplementary material for: Clinical analysis of secondary glaucoma in Central China
Source: Sci Rep. 2023 May 25;13:8439. doi: 10.1038/s41598-023-34872-8 (PMC10209582; doi:10.1038/s41598-023-34872-8)
Supplement: Supplementary file 1 — Supplementary Figures. [file 41598_2023_34872_MOESM1_ESM.docx]

# Supplementary Information

Clinical Analysis of Secondary Glaucoma in Central China

Qian Liu^1*^, Changgeng Liu^1^, Wenjun Cheng^1^, Xiaomei Feng^1^, Haijun Li^1^, Xiaoyuan Yang^1^, Yangzeng Dong^1^

^1^Henan Provincial People’s Hospital, Henan Eye Hospital, Henan Eye Institute, Zhengzhou University People’s Hospital, Zhengzhou, China


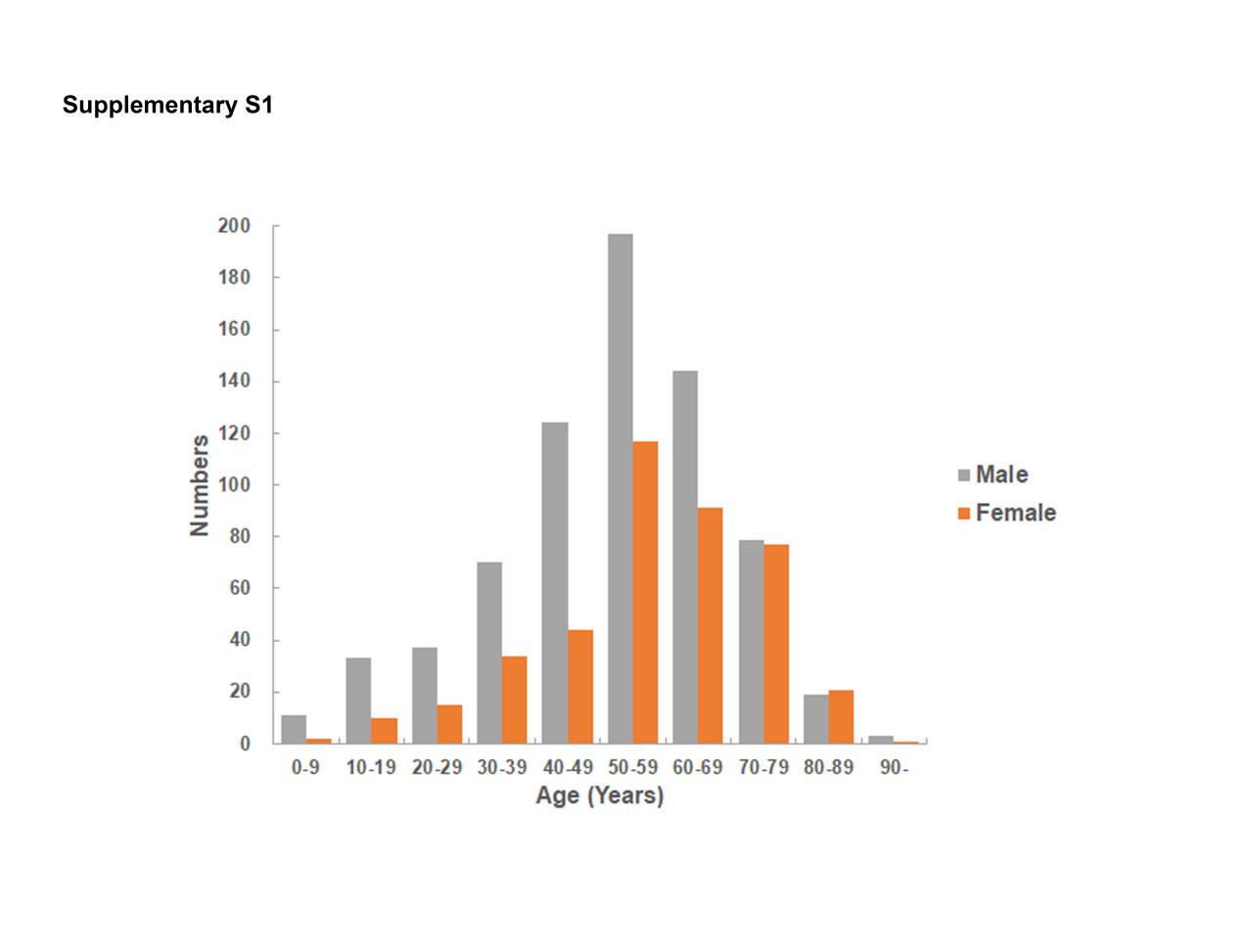


**Supplementary S1 Distribution of sex and age ranges among patients with secondary glaucoma.** Among male patients, the highest incidence of secondary glaucoma was found in the middle-aged and older groups (40–49, 50–59, 60–69 years). Among female patients, the highest incidence was found in older groups (50–59, 60–69, 70–79 years).


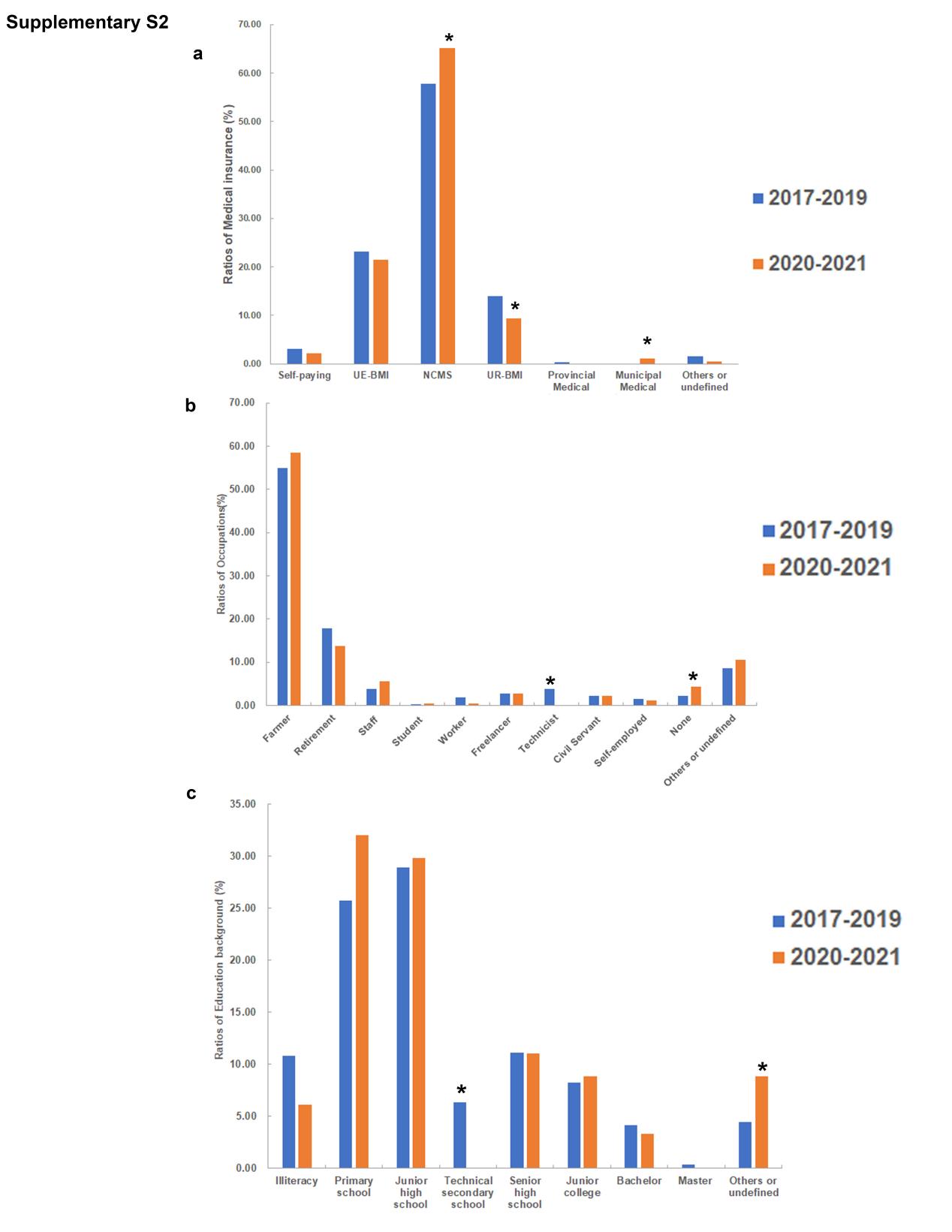


**Supplementary S2 Ratios of medical insurance, occupation, and educational background characteristics among patients with neovascularization-induced secondary glaucoma.** a) Ratios of medical insurance types among patients with neovascularization-induced secondary glaucoma. b) Ratios of occupations among patients with neovascularization-induced secondary glaucoma. c) Ratios of educational levels among patients with neovascularization-induced secondary glaucoma.

**
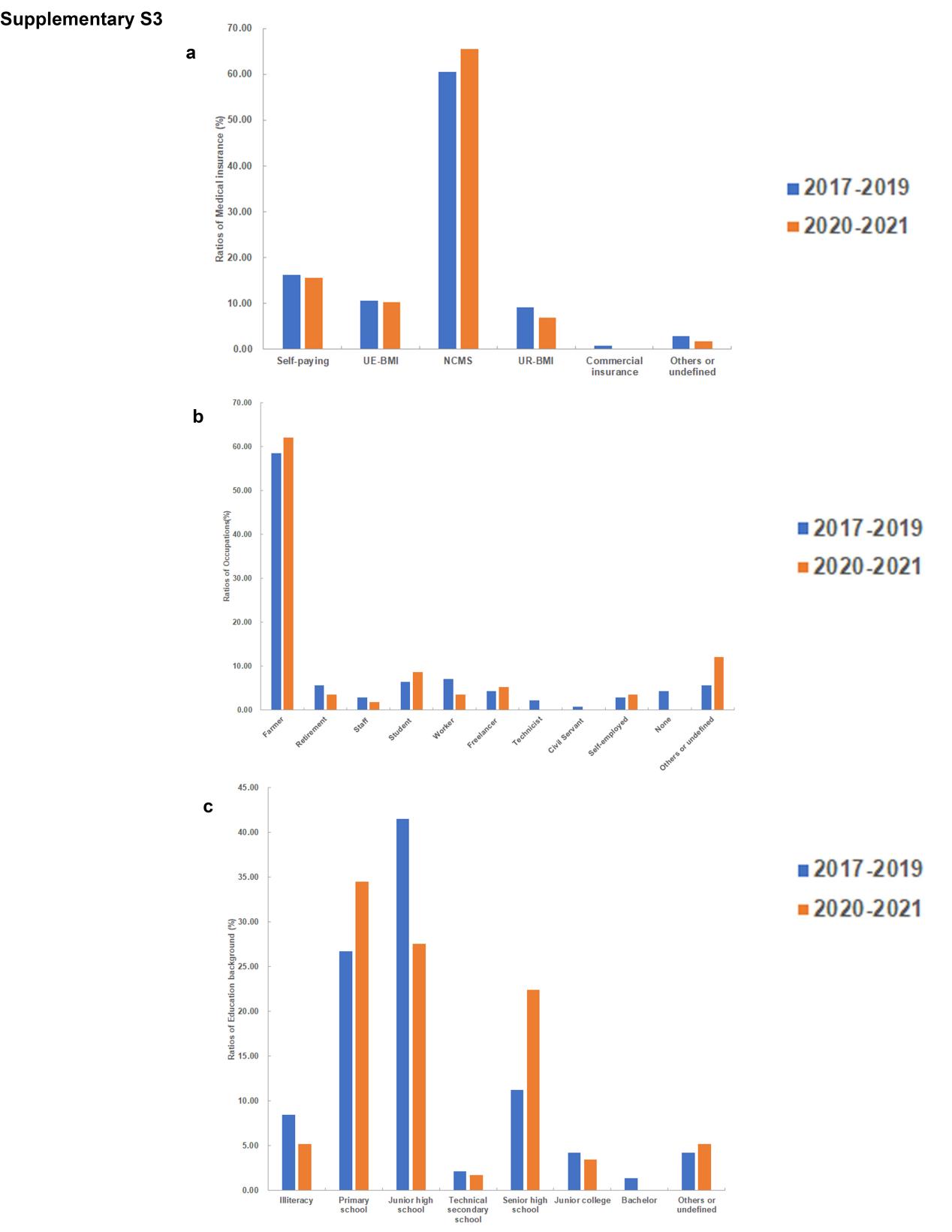
**

**Supplementary S3 Ratios of medical insurance, occupation, and educational background characteristics among patients with traumatic secondary glaucoma.** a) Ratios of medical insurance types among patients with traumatic secondary glaucoma. b) Ratios of occupations among patients with traumatic secondary glaucoma. c) Ratios of educational levels among patients with traumatic secondary glaucoma.
